# Stanford type A acute aortic dissection with proximal intimo-intimal intussusception: a case report and literature review

**DOI:** 10.1186/s13019-021-01581-0

**Published:** 2021-07-28

**Authors:** Hao Pan, Wei Sun

**Affiliations:** Department of Anesthesiology, Shandong Provincial Hospital Affiliated to Shandong First Medical University, No.324 Jingwu road, Jinan, 250021 Shandong China

**Keywords:** Acute aortic dissection, Intimo-intimal intussusception, Aortic regurgitation

## Abstract

**Background:**

Acute aortic circumferential dissection with proximal intimo-intimal intussusception is a rare and potentially lethal occurrence. We here report a case and review previous works to better understand this particular condition and help surgeons to determine accurate diagnosis and optimal intervention strategies by intraoperative transesophageal echocardiography (TEE).

**Case presentation:**

We report a case of a 46-year-old male who complained of sudden substernal chest pain. Stanford type A acute aortic dissection with proximal intimo-intimal intussusception was confirmed by contrast-enhanced computed tomography (CECT), transthoracic echocardiography (TTE), and TEE. We found the intimal flap prolapsed into the left ventricle outflow tract (LVOT), which caused severe aortic regurgitation (AR) and obstructed the ostia of the coronary arteries. Given the preexisting aneurysmal dilatation of aortic sinus and severity of aortic root and arch dissection, Bentall procedure and Sun’s procedure were performed for our patient.

**Conclusions:**

Intraoperative TEE used by anesthesiologists here played an increasingly valuable role in the determination of acute aortic dissection. Hence, it is necessary that TEE screening is routinely performed in patients with acute aortic dissection to provide valuable information for facilitating surgical strategies.

**Supplementary Information:**

The online version contains supplementary material available at 10.1186/s13019-021-01581-0.

## Background

Acute aortic circumferential dissection with proximal intimo-intimal intussusception is a rare and potentially lethal occurrence [1], capable of causing severe aortic regurgitation (AR) or malperfusion of coronary arteries [2]. Although several cases of this particular condition have been reported, it remains a rare event in clinical practice. Moreover, these patients usually have a rapid hemodynamic deterioration or collapse. Transesophageal echocardiography (TEE) is useful to determine the severity and mechanism of AR and provide a functional assessment of aortic valve [3]. Therefore, we reported this case and reviewed previous works to better understand this particular condition and help surgeons to determine accurate diagnosis and optimal intervention strategies by intraoperative TEE. Written consent was obtained from the patient for publication.

## Case presentation

A 46-year-old male presented to our emergency department with sudden substernal chest pain. He had a history of uncontrolled hypertension for 5 years. About 2 months earlier, he was admitted to the hospital for chest tightness and dyspnea after mild activity. The coronary angiography showed no significant stenosis or occlusion. Transthoracic echocardiography (TTE) revealed an aneurysmal dilatation of aortic sinus of 5.7 cm in diameter and ascending aorta of 4.2 cm in diameter, trileaflet aortic valve with aortic insufficiency causing moderate AR. The left ventricle (LV) was dilated to 6.9 cm in diameter. He declined surgical intervention in favor of medical therapy to improve his symptoms, and then he was discharged.

**Fig. 1:**
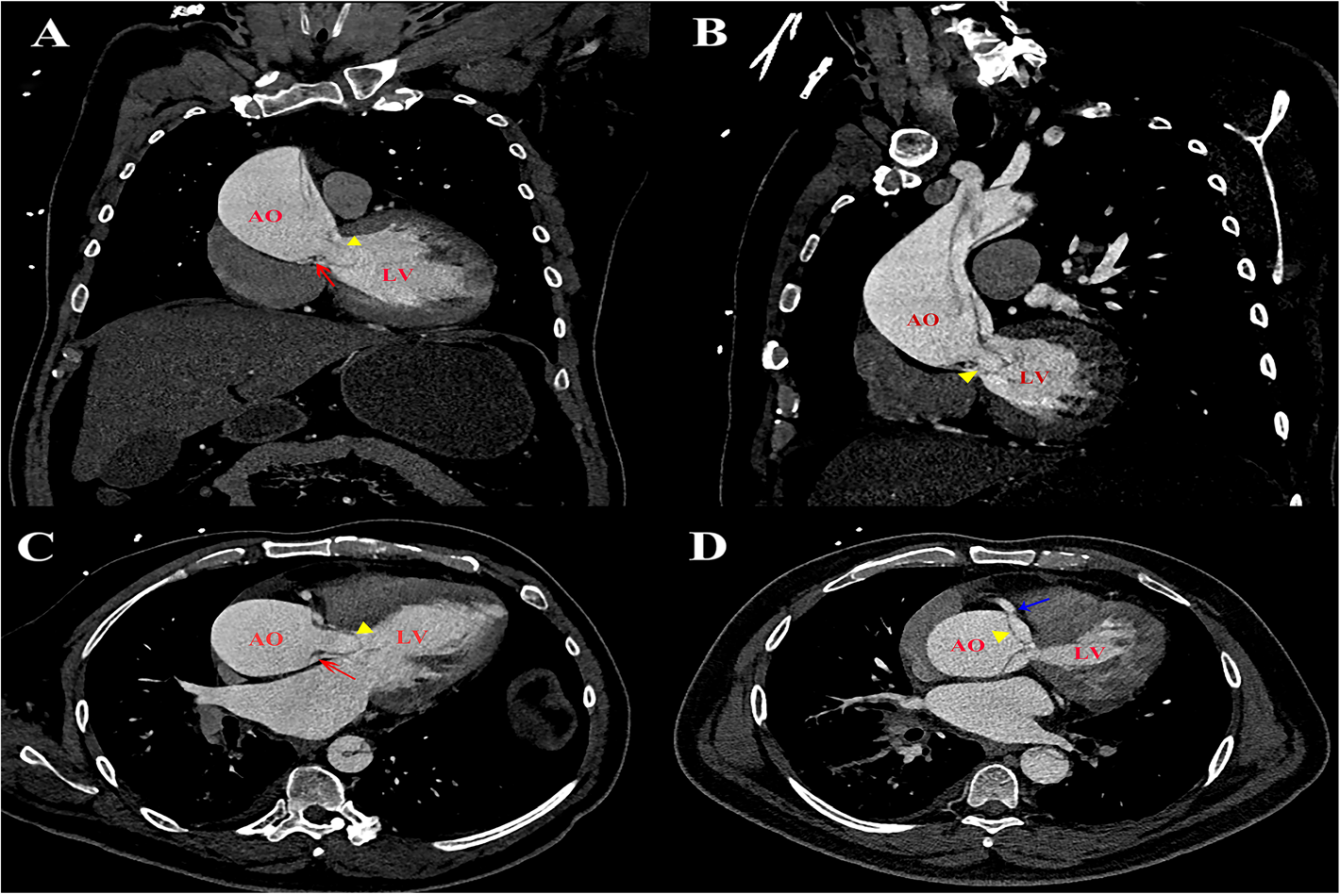
An intimal flap prolapsed into the left ventricular outflow tract in coronal view (**A**), sagittal view (**B**) and axial view (**C** and **D**) by CECT. Red arrow: aortic valve leaflet. Blue arrow: right coronary artery ostia. Yellow arrowhead: intimal flap. AO: aorta; LA: left atrium; LV: left ventricle; CECT: contrast-enhanced computed tomography

**Fig. 2:**
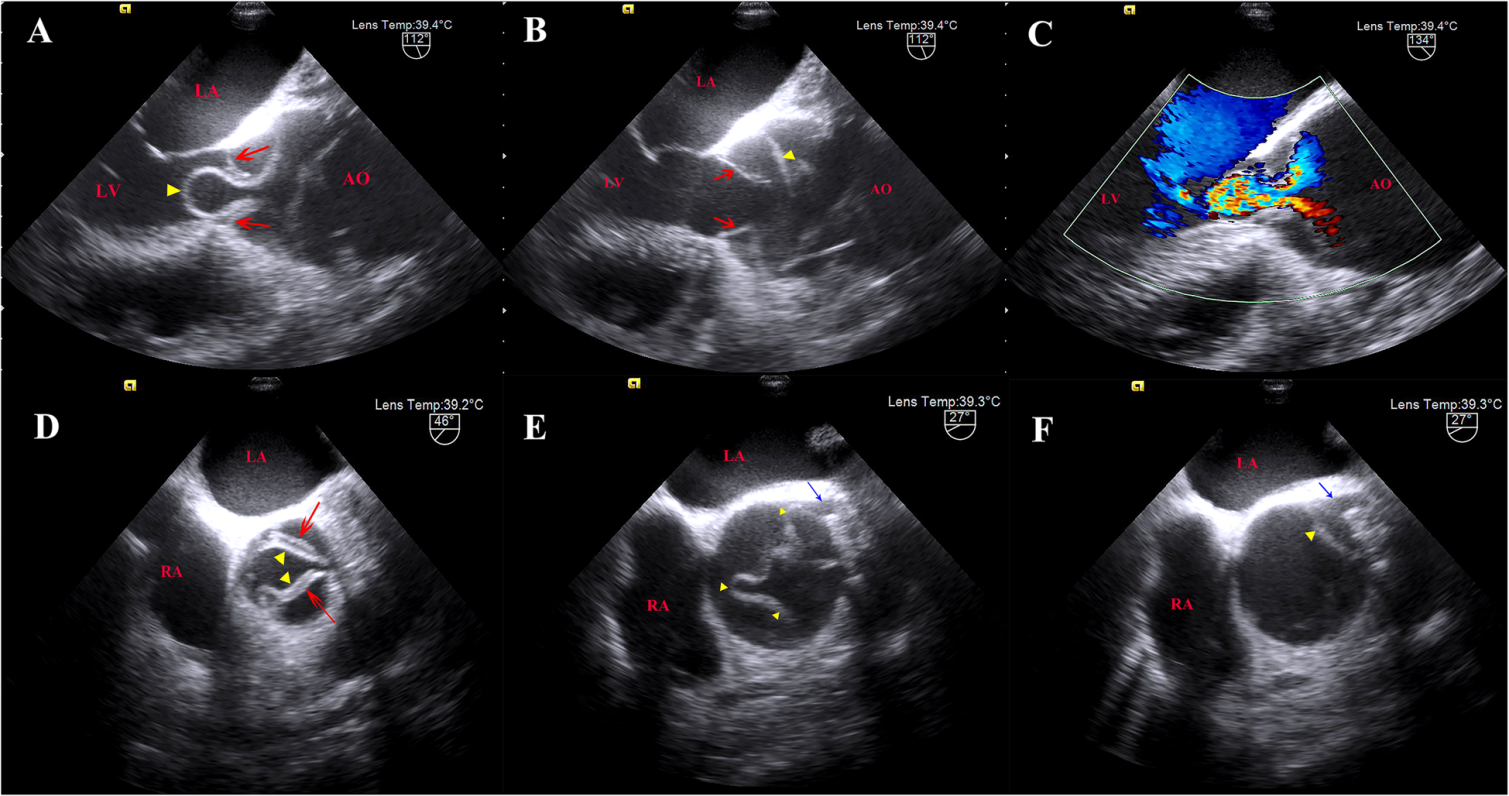
Transesophageal echocardiography showed views of the intimal flap in long axis (**A**: diastole, **B**: systole, **C**: diastole with color Doppler) and short axis (**D-F**). Red arrow: aortic valve leaflet. Blue arrow: left coronary artery ostia. Yellow arrowhead: intimal flap. LA: left atrium; RA: right atrium

On this admission, the patient suffered from chest pain for about 1 h. The blood pressure was 143/42 mmHg, and there was no difference between two arms. He was conscious and neurological examination showed no abnormalities. Electrocardiography showed ST segment depression in leads I, aVL and V4–6, and preoperative hsTnT level was elevated to 45.60 ng/L (under 14 ng/L is normal). Contrast-enhanced computed tomography (CECT) revealed a Stanford type A aortic dissection with circumferential dissection of ascending aorta, and the proximal intimal flap prolapsed into the left ventricle outflow tract (LVOT) (Fig. 1). The coronary arteries arising from the true aortic lumen were not involved in the dissection. The dissection extended distally to brachiocephalic trunk, bilateral common carotid artery, and left subclavian artery, and ended at right common iliac artery. TTE indicated aortic dissection with a proximal intimal flap moving in and out of the LVOT in synchronicity with the cardiac cycle causing severe AR. He was therefore scheduled for urgent surgical treatment. Intraoperative TEE further confirmed a balloon-like intimal flap prolapsing into LVOT during diastole, which interfered with aortic valve cusp mobility, causing severe AR (Fig. 2**,** Supplementary material video 1–4). However, regurgitant flow was concentrated in a balloon-like intimal flap, concealing the degree of preexisting aortic insufficiency. The LV was dilated to 7.0 cm in diameter with mild left ventricular wall hypertrophy, and mild left ventricular lateral wall motion abnormalities. The ascending aorta was severely involved by dissection and therefore, cardiopulmonary bypass was instituted by cannulation of right atrium and right axillary artery combined with right femoral artery. Surgical inspection confirmed that the intima of the ascending aorta had been torn away through almost all of its circumference and the dissection extended proximally to below the level of coronary arteries. However, the coronary arteries and aortic valve annulus were not involved with dissection. The aortic valve commissures were intact, and the aortic valve leaflets were degenerated with mild thickening and without prolapse and deformity. Bentall procedure (aortic valve replacement with ascending aorta graft implantation) and Sun’s procedure (total arch replacement using tetrafurcate graft with stented elephant trunk implantation) were performed. During hypothermic circulatory arrest, nasoparyngeal temperature was 25 °C and antegrade cerebral perfusion by combining right axillary artery with left common carotid artery was selected for cerebral protection. The total cardiopulmonary bypass time was 244 min, aortic cross-clamping time was 138 min and circulatory arrest time was 17 min. The surgery was uneventful, and the patient was discharged on the 7th postoperative day after a smooth recovery. Histopathological analysis revealed myxoid degeneration of aortic valve and ascending aortic wall.

## Discussion

The intimal disruption of acute aortic dissection is usually transverse and rarely exceeds half the circumference of the aorta [1]. In 1962, Hufnagel and Conrad first described this particular condition of acute aortic circumferential dissection with intimo-intimal intussusception [4]. Rosenzweig reported the incidence of this event to be under 2% in Stanford type A aortic dissection [5]. The intussusception of the mobile intimal flap might cause severe complications. Proximally, the intimal flap may prolapse retrograde into the LVOT, which can cause severe AR or obstruct the ostia of the coronary arteries during diastole [6]. Distally, the intimal flap may prolapse antegrade into aortic arch that can obstruct the aortic arch vessels affecting cerebral perfusion [6]. Nishioka et al. reported bidirectional intimo-intimal intussusception causing cerebral and myocardial ischemia and aortic regurgitation.

Given the serious hemodynamic instability and deterioration of these patients, timely diagnosis and urgent surgery are essential [7]. The most typical cause of coronary malperfusion is the retrograde aortic root dissection extending to ostia of coronary arteries [8]. The symptoms of patients with proximal intimo-intimal intussusception blocking the coronary ostia mimic those of acute coronary syndrome, so misdiagnosis, which is one of the primary preoperative risk factors, may be frequent [8]. Coronary angiography is usually selected for differential diagnosis. However, coronary angiography may fail to facilitate proper diagnosis because the intimal flap can obstruct the ostia of coronary arteries [2]. Although aortography can reveal aortic dissection, it cannot provide adequately accurate information for differentiating the intimal flap from aortic valve [6]. Nevertheless, there have been reports that angiography can reveal aortic dissection with an intimal flap prolapsing into LVOT during diastole [2]. Currently, multidetector row CT and electrocardiography gated CT can produce clear images and allow detection of aortic dissection with circumferential intimal flap prolapsing into LVOT during diastole and differentiate the intimal flap from aortic valve [6, 9]. Preoperative TTE is also a convenient and practical tool to determine aortic dissection with proximal intimo-intimal intussusception [10, 11]. TEE can detect continuous and real-time motion of the intimal flap, degree of blocking ostia of the coronary arteries, and degree of intussusception causing AR and function of the left ventricle [6, 12]. Intraoperative TEE is considered the most practical and reliable diagnostic modality for diagnosing acute aortic dissection with intimo-intimal intussusception [6]. Therefore, the causes of myocardial ischemia in our case were considered to be associated with coronary malperfusion of a prolapsed intimal flap obstructing the ostia of coronary arteries based on previous coronary angiography, CT, TTE, and TEE findings.

Severe AR due to prolapse of an intimal flap into LVOT of acute aortic dissection is a rare event. The hypothesis proposed by Chow et al. states that aortic root dilation may be the prerequisite condition of prolapsed intimal flap-induced AR, in this case resulting in the phenomenon of “regurgitation begets regurgitation” [3]. The characteristics of the patient in our case were consistent with this hypothesis. The structure and function of aortic valve were evaluated carefully in real time by midesophageal long axis and short axis views. However, the degree of preexisting aortic insufficiency was covered up by the prolapsed intimal flap-induced AR. As a result, surgeons are forced to inspect aortic valve directly in a state with aorta and heart empty and flaccid after commencing cardiopulmonary bypass [13]. Although experienced surgeons may determine whether the replacement or repair of aortic valve is or is not based on direct inspection alone of aortic valve, it is preferable for surgeons to evaluate the aortic valve in a physiologic state with aorta full and heart beating [13].

The structure and function of aortic valve in these patients with proximal intimo-intimal intussusception-induced AR are usually intact. In this way, aortic valve preservation may be feasible. Chow et al. reported that one aortic valve was preserved due to the absence of any prolapse or disruption of leaflets or commissures [3]. Ito et al. performed valve-sparing aortic root replacement for a patient with extremely localized aortic dissection with intimo-intimal intussusception [6]. Nakamura et al. also reported a case of aortic circumferential dissection with intimo-intimal intussusception that had not received aortic valve repair or replacement [14]. However, valve-sparing procedures prolong the operation, especially in those patients who also require complete arch repair, and it can also extend the time required for cardiopulmonary bypass [15]. The imperative objective in this surgical emergency of Stanford type A acute aortic dissection is to save patients’ lives. Although repair of aortic root and valve is safe, effective, and durable, the approach to surgical intervention should take into consideration factors such as patient presentation, echocardiographic assessment of proximal aorta, and surgical experience so as to reduce mortality and morbidity [16]. Contreras et al. stated their indications for aortic valve replacement as aortic root aneurysm, diseased/heavily calcified bicuspid aortic valve or aortic dissection involved in aortic annulus [17]. Only about 5% patients of Stanford type A aortic dissection needed aortic valve replacement, and another 5–10% could benefit from aortic valve resuspension in their institution [17]. Given the preexisting aneurysmal dilatation of aortic sinus and severity of aortic root and arch dissection, surgeon decided to perform aortic root and aortic valve replacement for our patient.

## Conclusion

Acute aortic circumferential dissection with intimo-intimal intussusception is a rare and serious event that can cause severe complications. Intraoperative TEE used by anesthesiologists played an increasingly valuable role in the determination of acute aortic dissection, mechanism of AR and evaluation of severity of AR. Therefore, it is necessary that TEE screening is routinely performed by anesthesiologists in patients with Stanford type A acute aortic dissection to provide valuable information for facilitating surgical strategies.

## Supplementary Information

**Additional file 1.**

**Additional file 2.**

**Additional file 3.**

**Additional file 4.**

## Data Availability

All data/files can be obtained from the corresponding author.

## Abbreviations

TEE: Transesophageal echocardiography
CECT: Contrast-enhanced computed tomography
CT: Computed tomography
TTE: Transthoracic echocardiography
LVOT: Left ventricle outflow tract
LV: Left ventricle
AR: Aortic regurgitation

## References

[1] Yavuz S, Elhan K, Eris C, Tugrul Goncu M (2003). Intimo-intimal intussusception: a rare clinical form of aortic dissection. Eur J Cardiothoracic Surg.

[2] Lajevardi SS, Sian K, Ward M, Marshman D (2012). Circumferential intimal tear in type a aortic dissection with intimo-intimal intussusception into left ventricle and left main coronary artery occlusion. J Thorac Cardiovasc Surg.

[3] Chow JL, Mariano ER, Liang D (2007). Transesophageal echocardiography assessment of severe aortic regurgitation in type a aortic dissection caused by a prolapsed circumferential intimal flap. J Cardiothorac Vasc Anesth.

[4] Hufnagel CA, Conrad PW (1962). Intimo-intimal intussusception in dissecting aneurysms. Am J Surg.

[5] Rosenzweig BP, Goldstein S, Sherrid M, Kronzon I (1996). Aortic dissection with flap prolapse into the left ventricle. Am J Cardiol.

[6] Ito Y, Nakamura Y, Kuroda M, Endo Y, Nakanishi Y, Hori T (2018). Valve-sparing aortic root replacement for extremely localized circumferential aortic dissection associated with intimo-intimal intussusception. Gen Thorac Cardiovasc Surg.

[7] Yamabi H, Imanaka K, Sato H, Matsuoka T (2011). Extremely localized aortic dissection and intussusception of the intimal flap into the left ventricle. Ann Thoracic Cardiovasc.

[8] Neri E, Toscano T, Papalia U, Frati G, Massetti M, Capannini G, Tucci E, Buklas D, Muzzi L, Oricchio L, Sassi C (2001). Proximal aortic dissection with coronary malperfusion: presentation, management, and outcome. J Thorac Cardiovasc Surg.

[9] Ko SM (2009). MDCT findings of acute aortic dissection with diastolic prolapse of the intimal flap into the left ventricle. Br J Radiol.

[10] Uçar O, Canbay A, Demirçelik B, Aydoğdu S (2010). Acute type a aortic dissection with diastolic prolapse of intimal flap into the left ventricle. Turk Kardiyoloji Dernegi Arsivi.

[11] Efem MS, Gur DO, Ersoz E (2012). Balloon-like intimal flap in the left ventricle. Eur Heart J.

[12] Yamashita Y, Nakagawa S, Kitamoto S, Sakamoto K, Horii T (2020). A case of circumferential type a aortic dissection with Intimo-intimal intussusception. Ann Vascular Dis.

[13] Movsowitz HD, Levine RA, Hilgenberg AD, Isselbacher EM (2000). Transesophageal echocardiographic description of the mechanisms of aortic regurgitation in acute type a aortic dissection: implications for aortic valve repair. J Am Coll Cardiol.

[14] Nakamura R, Honda K, Yuzaki M, Nishimura Y (2020). Severe aortic regurgitation with intimal intussusception secondary to Debakey type I aortic dissection. Echocardiography (Mount Kisco, NY).

[15] Parikh N, Trimarchi S, Gleason TG, Kamman AV, di Eusanio M, Myrmel T, Korach A, Maniar H, Ota T, Khoynezhad A, Montgomery DG, Desai ND, Eagle KA, Nienaber CA, Isselbacher EM, Bavaria J, Sundt TM, Patel HJ (2017). Changes in operative strategy for patients enrolled in the international registry of acute aortic dissection interventional cohort program. J Thorac Cardiovasc Surg.

[16] Patel PA, Bavaria JE, Ghadimi K, Gutsche JT, Vallabhajosyula P, Ko HA, Desai ND, Mackay E, Weiss SJ, Augoustides JGT (2018). Aortic regurgitation in acute type-a aortic dissection: a clinical classification for the perioperative Echocardiographer in the era of the functional aortic annulus. J Cardiothorac Vasc Anesth.

[17] Contreras V, Sheinbaum R, Tran S, Zaki J, Moise O (2018). Aortic regurgitation in acute type a dissection. J Cardiothorac Vasc Anesth.

